# Functionality of Dengue Virus Specific Memory T Cell Responses in Individuals Who Were Hospitalized or Who Had Mild or Subclinical Dengue Infection

**DOI:** 10.1371/journal.pntd.0003673

**Published:** 2015-04-13

**Authors:** Chandima Jeewandara, Thiruni N. Adikari, Laksiri Gomes, Samitha Fernando, R. H. Fernando, M. K. T. Perera, Dinuka Ariyaratne, Achala Kamaladasa, Maryam Salimi, Shamini Prathapan, Graham S. Ogg, Gathsaurie Neelika Malavige

**Affiliations:** 1 Centre for Dengue Research, Department of Microbiology, Faculty of Medical Sciences, University of Sri Jayawardanapura, Nugegoda, Sri Lanka; 2 MRC Human Immunology Unit, Weatherall Institute of Molecular Medicine, Oxford NIHR Biomedical Research Centre and University of Oxford, Oxford, United Kingdom; 3 Department of Dermatology, Churchill Hospital, Oxford, United Kingdom; University of Rhode Island, UNITED STATES

## Abstract

**Background:**

Although antibody responses to dengue virus (DENV) in naturally infected individuals have been extensively studied, the functionality of DENV specific memory T cell responses in relation to clinical disease severity is incompletely understood.

**Methodology/Principal findings:**

Using ex vivo IFNγ ELISpot assays, and by determining cytokines produced in ELISpot supernatants, we investigated the functionality of DENV-specific memory T cell responses in a large cohort of individuals from Sri Lanka (n=338), who were naturally infected and were either hospitalized due to dengue or had mild or sub clinical dengue infection. We found that T cells of individuals with both past mild or sub clinical dengue infection and who were hospitalized produced multiple cytokines when stimulated with DENV-NS3 peptides. However, while DENV-NS3 specific T cells of those with mild/sub clinical dengue infection were more likely to produce only granzyme B (p=0.02), those who were hospitalized were more likely to produce both TNFα and IFNγ (p=0.03) or TNFα alone.

We have also investigated the usefulness of a novel T cell based assay, which can be used to determine the past infecting DENV serotype. 92.4% of DENV seropositive individuals responded to at least one DENV serotype of this assay and none of the seronegatives responded. Individuals who were seronegative, but had received the Japanese encephalitis vaccine too made no responses, suggesting that the peptides used in this assay did not cross react with the Japanese encephalitis virus.

**Conclusions/significance:**

The types of cytokines produced by DENV-specific memory T cells appear to influence the outcome of clinical disease severity. The novel T cell based assay, is likely to be useful in determining the past infecting DENV serotype in immune-epidemiological studies and also in dengue vaccine trials.

## Author Summary

Although dengue viral infections cause severe clinical disease, the majority of individuals infected with the dengue virus (DENV) develop asymptomatic infection. The function of DENV specific memory T cells in relation to past clinical disease severity is incompletely understood. In this study, we sought to investigate the function of DENV specific memory T cell responses in a large cohort (n = 338) of individuals who were naturally infected with the DENV but developed varying severity of clinical disease. We found that T cells of individuals who were hospitalized due to dengue and those with mild/sub clinical dengue infection produced multiple cytokines when stimulated with DENV-NS3 peptides. In addition, we have also validated a novel T cell based assay, which can be used to determine the past infecting DENV serotype. We found that 92.4% of DENV seropositive individuals responded to at least one DENV serotype of this assay and none of the seronegatives responded. Moreover, the peptides used in this assay did not cross react with Japanese encephalitis virus. Therefore, this assay is likely to be useful in determining the past infecting DENV serotype in immune-epidemiological studies and also in dengue vaccine trials.

## Introduction

Dengue viral infections are one of the most important emerging virus infections in the world [1] causing 390 million dengue infections annually, of which 96 million are clinically apparent [2][3]. 70% of infections occur in Asia and as a result of the high disease burden, dengue has been declared a priority infection by the WHO, UNICEF and World Bank [4]. Currently there are no effective antiviral drugs to treat acute infection, nor a licensed vaccine to prevent infection.

The main hurdle in developing a safe and effective vaccine has been our poor understanding of the complex nature of the protective immune response in acute dengue infection and the presence of four dengue virus (DENV) serotypes that are highly homologous[5]. One of the main concerns, especially after the results of the recent phase IIb and a phase III dengue vaccine clinical trials has been the poor understanding of T cell correlates of protection [6–8]. Although DENV-specific, highly cross reactive T cells were believed to contribute to severe clinical disease [9–11], [12] more recent data suggest that T cell responses are likely to be protective [13, 14]. Extensive analysis of T cell responses from a large cohort of naturally infected individuals showed that DENV-specific T cell responses of higher magnitude, and which were multifunctional were directed towards HLA alleles associated with reduced disease susceptibility [13]. Polyfunctional T cell responses have shown to be protective in many virus infections [15–18]. However, in dengue infection, DENV-specific memory T cell responses are thought to produce highly pro-inflammatory cytokines and are also thought to be sub optimal in clearing the virus in secondary dengue infection[10, 12, 19]. Therefore, as the functionality of DENV-specific memory T cell responses can possibly influence the outcome of subsequent infection with DENV, we proceeded to investigate the functionality of DENV-specific T cell responses in a large cohort of naturally infected individuals who were either hospitalized due to dengue or who had mild/subclinical past infection.

Although secondary dengue infections are known to associate with more severe disease [20, 21], the majority of both primary and secondary dengue infections lead to asymptomatic disease [21, 22]. Therefore, in order to understand how previous dengue infections contribute to clinical disease severity, it would be crucial to determine the past infecting serotype and subsequent immune responses in naturally infected individuals who develop symptomatic and asymptomatic infection. Currently the most widely used method to determine past infecting serotype in DENV-seropositive individuals is the plaque reduction neutralization assay (PRNT)[23, 24]. However, there have been many concerns regarding the variability of the PRNT results, especially in those who had been infected with multiple DENV-serotypes [25–28]. In addition, a recent study showed that individuals who demonstrated high neutralizing antibodies for a particular DENV serotype (which suggested past infection with that serotype according to the PRNT) later went on to develop DHF when infected with that particular serotype[28]. Collectively, these data suggests that the PRNT may not be the most suitable method for determining the past infecting serotype and other methods should be developed. In this study, we further investigated the use of a previously described panel of serotype specific peptides, from highly conserved regions of the DENV in determining the past infecting serotype [29]. We found that DENV-seronegative individuals did not generate any responses to these peptides, while 92.4% of DENV-seropositive individuals responded to peptides of at least one DENV serotype. In addition, during the study period, those who were hospitalized due to a primary dengue infection (who were seronegative at the time of recruitment) responded to peptides of only a single DENV-serotype. Therefore, as this novel T cell based assay appears to detect DENV-serotype specific responses, it has a potential to be used in dengue vaccine trials to determine the past infecting DENV-serotypes and thus pre-existing DENV immunity.

## Materials and Methods

### Study area and participants

The study included 338 individuals attending the Family Practice Center, which is a primary health care facility of the University of Sri Jayewardenepura, Sri Lanka providing primary health care to over 2000 individuals living in the suburban areas of the Colombo district. Individuals registered at the Family Practice Center between the ages of 6 to 80 years were invited to participate in this study and were recruited following informed written consent. Ethical approval was granted by Ethical Review Committee of the University of Sri Jayawardanapura.

Blood samples were obtained and an interviewer administered questionnaire was used to record demographic details. We had access to detailed medical records of all participants which included information regarding all hospital admissions, presence of co-morbid factors, drug history and vaccination records. None of the participants reported a past JEV infection. According to classification of WHO guidelines 2011[4], of the 54/338 of those who were hospitalized due to dengue infection, 8 (14.8%) were diagnosed with DF and 46 (85.2%) had DHF. The Ministry of Health of Sri Lanka, has laid down criteria for admission of patients with suspected dengue infection to hospital and hospitals follow these criteria for admission. Therefore, any individual with a suspected dengue infection, who presents with any of the clinical features listed below, is admitted to hospital, following a clinical assessment carried out by the medical officers at the out-patient department.

The criteria for admission are as follows:

- A person with fever and a platelet count <100,000/mm^3^
- Presence of dengue warning signs such as abdominal pain or tenderness and persistent vomiting
- Patients with any clinical signs of plasma leakage such as the presence of pleural effusions, ascites or the presence of mucosal bleeding, lethargy and restlessness,
- liver enlargement >2 cm
- An increase in the haematocrit with concurrent rapid decrease in platelet count in a full blood count

At the time of recruitment of the study participants, they were categorized as having hospitalized dengue based on the diagnosis card given by the hospitals. This diagnosis card carried information such as whether the patient has DHF or DF and the laboratory investigations while in hospital. As dengue antibody assays or dengue NS1 antigen assays are not routinely done in government hospitals, the dengue NS1 antigen test results or the dengue antibody test results were only available for some of these 54 patients. The other patients were diagnosed as having either DF or DHF based on clinical features. The individuals who had been hospitalized due to dengue were recruited at least 6 months after the episode of their hospitalization. The time of infection of those with mild/subclinical dengue infection was not known.

### Peptides

A panel of 17, 20mer peptides were synthesized in-house in an automated synthesizer using F-MOC chemistry. These peptides have been previously described [29] and were found to be serotype specific and originating from highly conserved regions of the four DENVs. There were four peptides specific to DENV-1, five specific to DENV-2, four specific for DENV-3 and four specific for DENV-4. Of these 17 peptides, except for 2 peptides which induced both CD4+ and CD8+ T cell responses, all other peptides induced CD4+ T cell responses. Dengue NS3 peptides were again 20 mer peptides overlapping by 10 amino acids, which spanned the whole length of the DENV-3 NS3 protein. The synthetic NS3 20mer peptides were pooled together to represent the whole NS3 protein. The FEC control peptides that were used contain a panel of 23, 8–11 amino acid CD8+ T cell epitopes of Epstein Barr virus (EBV), Flu and CMV viruses and have been used as quality control in ELISpot assays [30].

### *Ex vivo* ELISpot assays

*Ex vivo* Elispot assays were performed as previously described [31, 32] in 242 individuals with mild/sub clinical dengue infection, 54 individuals with past hospitalized dengue and 42 seronegative volunteers (a total of 338 individuals). Briefly ELISpot plates (Millipore Corp., Bedford, Massachusetts, USA) were coated with anti-human IFNγ antibody (Mabtech AB, Nacka, Sweden) overnight. DENV-NS3 overlapping peptides and FEC peptides were added at a final concentration of 10 μM as previously described [12, 33]. The live attenuated SA-14-14-2 JEV vaccine was used at a PFU concentration of ≥5.4 log PFU/ELISpot well. PHA was used as a positive control and an irrelevant peptide (SARS 20 mer peptide) was included as a negative control in addition to background control (cells with media). The spots were enumerated using an automated ELISpot reader (AID, Germany). Background (cells with media) was subtracted and data expressed as number of spot-forming units (SFU) per 10^6^ PBMC. The DENV-3 NS3 peptides were tested in a single pool of peptides (all the 20mer peptides were pooled together) in duplicate. Mean of the response is indicated in the data points as SFC/million PBMC. All peptides that induced an IFN-γ response of more than mean ±3 standard deviations of the irrelevant peptide were considered positive.

### Cultured ELISpot assays

Cultured ELISpot assays were performed on 82 individuals with mild/sub clinical dengue infection, in 23 who were hospitalized due to dengue and 30 seronegative individuals as previously described [29, 31, 32]. All peptides that induced an IFN-γ response of more than mean±3 standard deviations of the irrelevant peptide were considered positive.

### Serology

All individuals were tested by an indirect dengue IgG capture ELISA (Panbio) for the qualitative detection of IgG antibodies to DENV. Pan Bio units were calculated according to the manufacturer’s instructions and accordingly, Pan Bio units of >11 were considered positive, 9–11 was considered equivocal and <9 was considered negative. JE Detect IgG ELISA (Inbios) was used for the detection of JEV specific-IgG antibodies. Calculation of the immune status ratio (ISR) was done according to the manufacturers’ instructions and accordingly an ISR of >5 was considered positive; an ISR of 2–5 equivocal and an ISR of <2 negative.

### Quantification of cytokines

Quantitative cytokine assays were done in duplicate on ex vivo ELISpot culture supernatants stimulated with DENV-NS3, JEV, FEC and the wells that only contained media. Quantitative ELISA was done for granzyme B (BioLegend, USA), TNFα (BioLegend, USA) and IL 2 (Mabtech, Sweden) according to manufactures instructions. Accordingly 2.4 pg/mL ≥ of granzyme B production, 3.5 pg/ml ≥ TNF-α production and 0.4 pg/mL ≥ of IL2 production was considered positive.

### Statistical analysis

PRISM version 6 was used in statistical analysis. As the data were not normally distributed, differences in means were compared using the Mann-Whitney U test (two tailed). To compare means of three or more variables, Kruskal-Wallis test was used. Degree of associations between hospitalized dengue infection, mild/sub clinical dengue infection and type of cytokines produced by the PBMCs and response to serotype specific peptides of three or more DENVs or 4> DENVs was expressed as the odds ratio (OR), which was obtained from standard contingency table analysis by Haldane’s modification of Woolf’s method. Chi Square tests or the Fisher’s exact test was used to determine the p value.

## Results

Of the 338 individuals who were recruited, 182 (53.8%) were females and 156 (46.1%) were males. The mean age was 39.86 (95% CI 35–44.72). 296/338 (87.6%) individuals were seropositive for dengue infection. 242/296 (81.8%) were considered to be have had a mild/subclinical past dengue infection, as they had never been hospitalized due to a febrile illness and therefore, are most likely to have had mild clinical disease or were asymptomatic. Of the 54 individuals who had been hospitalized due to a dengue infection, 8 (14.8%) had DF and 46 (85.2%) had DHF. The mean ages of those who were hospitalized due to dengue and who had mild/sub clinical dengue were similar (43.4 vs. 43.3 years) and the gender distribution of the two groups were similar (females 55.6% vs 53.3%).

### Functionality of the T cell responses in those who were hospitalized and those who had subclinical infection

**Fig 1:**
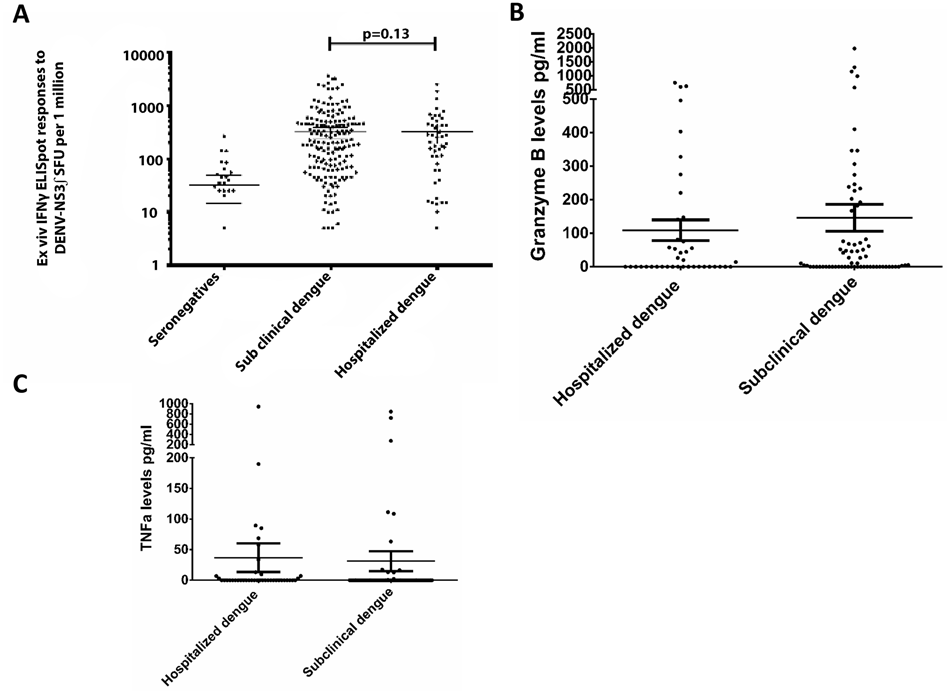
Cross reactive dengue-specific immune responses and disease severity. A: Circulating NS3-specific IFNγ ex vivo ELISpot responses were measured in individuals who were hospitalized due to dengue and in those with past mild/sub clinical dengue infection. B: Granzyme B production by PBMCs from individuals who were hospitalized due to dengue and who had a past mild/sub clinical dengue infection, following stimulation with DENV-NS3 overlapping peptides. C: TNFα production by PBMCs from individuals who were hospitalized due to dengue and those with past mild/sub clinical dengue infection following stimulation with DENV-NS3 overlapping peptides.

Although cross reactive T cells have been implicated in causing severe dengue [10, 12, 34], more recent studies done in individuals naturally infected with the DENV suggest that DENV-NS3 specific T cell responses could be protective [13, 14, 35]. However, the frequency and functionality of memory T cell responses in these cohorts were not assessed based on past clinical disease severity. As DENV-NS3 has shown to be one of the most immunodominant DENV nonstructural proteins and since it was shown to induce predominantly CD8+ T cell responses, we proceeded to use one pool of DENV-NS3 overlapping peptides to determine the functionality of T cell responses [32, 34]. Our initial aim was to determine if the frequency of IFNγ NS3 specific memory T cell responses were different in those with mild/sub clinical dengue infection when compared to those who were hospitalized due to dengue. We found that individuals who were hospitalized due to dengue did not have a significantly higher (p = 1.0) frequency of NS3-specific IFNγ ELISpot responses when compared to those with mild/sub clinical dengue infection (Fig 1A). Of the 54 individuals who were hospitalized 47 (87.0%) had a positive IFNγ ELISpot response to the NS3 peptides, while 170 (70.2%) of those with mild/sub clinical dengue infection were positive. Therefore, it appears that although the magnitude of IFNγ production by NS3 specific T cells were not different in those who were hospitalized and in those with mild/sub clinical dengue infection, DENV-NS3 specific T cells of those who were hospitalized were more likely to produce IFNγ (p = 0.02, odds ratio = 2.7, 95% CI 1.2 to 6.3).

As production of a single cytokine does not indicate the functionality of DENV-NS3 specific T cells in those with varying severity of past infection, we next proceeded to investigate the relationship between functionality of DENV-NS3 specific memory T cell responses and past clinical disease severity. In order to characterize the functionality of DENV-NS3 specific memory T cells, we determined cytokine profiles of IFNγ, TNFα and granzyme B in individuals who were hospitalized and those with mild/sub clinical dengue infection. As determining the polyfunctionality of DENV-NS3 specific T cells at an individual cell level was beyond the scope of this study, we used ELISpot culture supernatants to determine cytokine production by antigen specific T cells. From our cohort, ELISpot culture supernatants of PBMCs stimulated with DENV-NS3 peptides of 70 individuals with mild/sub clinical dengue infection and 41 individuals who were hospitalized were further assessed for production of the above cytokines. However, DENV-NS3 specific T cells of only 7 (10%) of those with past mild/sub clinical dengue infection and 7 (17.1%) of those who were hospitalized, produced 3 cytokines (IFNγ, TNFα and granzyme B, Table 1). Although we also investigated IL-2 production by DENV-NS3 specific T cells in the ELISpot supernatants, there was negligible amounts of IL-2 production.

**Table 1:** Number and percentage of individuals with mild/sub clinical dengue infection and those who were hospitalized due to dengue who produced of different cytokines when PBMCs were stimulated DENV-NS3 overlapping peptides and JE Live vaccine.

	Sub clinical dengue infection	Hospitalized dengue	P value
	N = 70 (%)	N = 41 (%)	
**Responses to DENV-NS3**			
IFNγ only	17 (24.3)	10 (24.4)	1
TNFα only	2 (2.9)	0 (0)	0.53
Granzyme B only	9 (12.9)	0 (0)	0.02*
IFNγ and TNFα only	4 (5.7)	8 (19.5)	0.03*
IFNγ and Granzyme B only	24 (34.3)	12 (29.3)	0.83
TNFα and Granzyme B only	7 (10)	4 (9.8)	1
All 3 cytokines	7 (10)	7 (17.1)	0.37
**Responses to JE live vaccine**			
IFNγ only	6 (8.6)	2 (4.9)	0.47
TNFα only	7 (10)	3 (7.3)	0.74
Granzyme B only	9 (12.9)	2 (4.9)	0.2
IFNγ and TNFα only	11 (15.7)	7 (17.1)	1
IFNγ and Granzyme B only	15 (21.4)	10 (24.4)	0.81
TNFα and Granzyme B only	7 (10)	8 (19.5)	0.4
All 3 cytokines	15 (21.4)	9 (21.9)	1

DENV-NS3 specific memory T cell responses of those who were hospitalized were significantly more likely to produce IFN γ and TNF α in the absence of granzyme B when compared to those with past mild/sub clinical dengue infection (p = 0.03, odds ratio = 4, 95% CI 1.1 to 14.3). In addition, DENV-NS3 specific T cells of 12.9% of those with mild/sub clinical dengue infection produced granzyme B only, whereas granzyme B only producing DENV-NS3 specific T cells were not detected in any of those who were hospitalized due to dengue (p = 0.02, odds ratio = 0.08, 95% CI 0.004 to 1.4). Therefore, production of granzyme B alone by DENV-NS3 specific T cells appeared to be associated with the occurrence of mild/subclinical infection.

The functionality of JEV specific memory T cell responses were also assessed in those with past hospitalized and mild/sub clinical dengue infection, as memory T cell responses to other closely related cross reactive flavi-viruses could influence the clinical disease outcome. Although, significant differences in the functionality of JEV-specific memory T cell responses were not observed in these two group of individuals, those with past mild/sub clinical dengue infection were more likely to produce granzyme B alone in response to JEV (Table 1).

Following analysis of types of cytokine production from PBMCs when stimulated with DENV-NS3, we proceeded to determine any differences in the quantity of cytokine production. There was no difference in quantity of granzyme B produced (p = 0.5) in those who were hospitalized due to dengue (mean 108.9; SD±196.5 pg/ml) when compared to those who had past mild/subclinical dengue (mean 146.3, SD±335.6 pg/ml) (Fig 1B). Similarly, no difference was seen (p = 0.1) in the quantity of TNFα production in those who were hospitalized due to dengue (mean 36.9, SD±149.7 pg/ml), when compared to those with past mild/subclinical dengue (mean 31.2, SD±135.7 pg/ml (Fig 1C).

### Investigation the use of a T cell based assay to determine serotype-specific immune responses

Severe dengue infection such as DHF is known to associate with secondary dengue infections [20, 36, 37] possibly due to the presence of cross reactive DENV-specific antibody and T cell responses [10, 38]. However, sub clinical dengue infection is also known to occur in those with secondary dengue infection [21]. Therefore, we next proceeded to determine if the number and the type of the past infecting DENV-serotype contributed to disease severity.

We had previously described a novel T cell assay [29], which uses several serotype specific peptides from highly conserved regions of the four DENVs. In this assay 4–5 peptides representing each DENV serotype are used to determine the serotype-specific T cell responses [29]. Therefore, as an initial step, we proceeded to determine the usefulness of these peptides in determining the past infecting serotype in a large cohort of individuals [29]. In order to further determine the usefulness of this assay we performed cultured ELISpot assays in 135 individuals in our study cohort. Of these 135 individuals, 30 of them were seronegative for the DENV, 23 individuals had been hospitalized due to dengue infection and 82 were seropositive but had sub clinical dengue infection.

None of the DENV-seronegative individuals (n = 30) responded to any of the serotype-specific peptides of the four DENV serotypes. Of these 30 seronegative individuals, 14/30 (46.7%), had received the JE vaccine. Despite been vaccinated with JE, they still not did respond to any of these DENV peptides, suggesting that these peptides are specific for dengue and do not cross react with JE specific T cells. 21/23 (91.3%) individuals with past hospitalized dengue and 76/82 (92.7%) of those with past mild/sub clinical dengue infection responded to at least one peptide of a DENV serotype. 37/105(35.2%) of seropositive individuals responded to peptides of two serotypes, 7/105(6.67) responded to peptides of three DENV serotypes of, 2/105 ((1.9%) have responded to peptides of all DENV serotypes

During the follow up a period of 15 months of the initial cohort of, 12/135 individuals in whom we had performed cultured ELISpot responses, developed a dengue infection requiring hospitalization. 7 of them were previously seronegative and 5 individuals were seropositive at the time of recruitment, but only responded to peptides of one serotype in this assay (Table 2). The decision to hospitalize these 12 patients was based on the criteria laid down by the Ministry of Health, Sri Lanka. In these 12 patients, dengue IgM and IgG was positive in all 5 patients with an acute secondary dengue infection and dengue-IgM was positive in all 7 who developed an acute primary dengue infection. The antibody tests carried out in hospital was reconfirmed by us, as we too bled the patients on day 21 following acute infection and performed dengue IgM and IgG antibodies. One of the previously seronegative individuals developed 2 episodes of hospitalized dengue infection. During the first dengue infection, only DENV-specific IgM was positive suggestive of a primary dengue infection. During the second episode of DHF, both DENV-specific IgM and IgG were positive, suggestive of a secondary dengue infection. Cultured ELISpot assays were carried out in 7 of the seronegative individuals and 5 of the previously seropositive individuals after the episode of DHF. 4/7 of the seronegative individuals responded to at least 1 peptide of the DENV-1 serotype, one responded to peptides of DENV-3 and one responded to peptides of DENV-4. Of the 5 dengue seropositive individuals who developed DHF, all had previously responded to only one serotype. Following the episode of DHF, all 5 responded to an additional DENV serotype and again it was found that 4/5 of them responded to at least one peptide of DENV1 (Table 2). This suggests that 8/12 of these individuals were likely to have been infected with DENV1 serotype, which accounted for >90% of infections during the study period (personal communication from Dr. Hasitha Tissera, Sri Lanka Epidemiology unit, Ministry of Health). The seronegative individual had two infections responded to two different serotypes (DENV-3 and DENV-1).

**Table 2:** Responses to the DENV serotype specific peptides of the 12 individuals at the time of recruitment and after they developed an episode of DHF during the study period.

Sample	Serial number	Type of infection	Positive responses at time of recruitment	SFC/ 1 million cells	DENV antibody titre (pre infection) Panbio unit	Positive responses following an episode of DHF during study period	SFC/ 1 million cells	Negative responses following an episode of DHF during study period	DENV antibody titre (post infection)
1	DW 1004	secondary	DENV3-21	737.5	25.35	DENV3-21; DENV1-20, DENV1-1	675	DENV 1–11, DENV 1–16,DENV 2–1, DENV 2–11, DENV 2–17, DENV 2–18, DENV 2–33, DENV 3–3, DENV 3–11, DENV 3–28, DENV 4–5, DENV 4–10, DENV 4–12, DENV 4–19	42.71
							2218.8		
							650.5		
2	DW 130	secondary	DENV3-28, DENV3-21	787.5	38.31	DENV3-28, DENV3-21; DENV1-20	687.5	DENV1-1,DENV 1–11, DENV 1–16,DENV 2–1, DENV 2–11, DENV 2–17, DENV 2–18, DENV 2–33, DENV 3–3, DENV 3–11, DENV 4–5, DENV 4–10, DENV 4–12, DENV 4–19	43.62
				450			378.5		
							787.5		
3	DW 466	secondary	DENV4-5, DENV4- 19	375	42.96	DENV4-5, DENV4-19; DENV3-3, DENV3-28	250	DENV1-1,DENV 1–11, DENV 1–16, DENV 20, DENV 2–1, DENV 2–11, DENV 2–17, DENV 2–18, DENV 2–33, DENV 3–11, DENV 3–21, DENV 4–12, DENV 4–19	51.38
				450			387.5		
							687.5		
							975		
4	DW 641	secondary	DENV3-3, DENV3-28	312.5	36.84	DENV3-3, DENV3-28; DENV1-1, DENV1-11	300	DENV 1–16, DENV 20, DENV 2–1, DENV 2–11, DENV 2–17, DENV 2–18, DENV 2–33, DENV 3–11, DENV 3–21, DENV 4–5, DENV 4–10, DENV 4–12, DENV 4–19	38.98
				450			375		
							987.5		
							675		
5	DW 1041	secondary	DENV3-3, DENV3- 11, DENV3-21	162.5	22.03	DENV3-3, DENV3- 11, DENV 3–21; DENV- 1, DENV1-11	175	DENV 1–16, DENV 20, DENV 2–1, DENV 2–11, DENV 2–17, DENV 2–18, DENV 2–33, DENV 3–28. DENV 4–5, DENV 4–10, DENV 4–12, DENV 4–19	33.22
				137.5			162.5		
				250			187.5		
							375		
							450		
6	DW 1071	primary	Nil	0	1.53	DENV 4–5, DENV 4–12, DENV 4–19	812.5	DENV 1–1,DENV 1–11, DENV 1–16, DENV 1–20, DENV 2–1, DENV 2–11, DENV 2–17, DENV 2–18, DENV 2–33, DENV 3–3, DENV 3–11, DENV 3–21 DENV 3–28, DENV 4–19	49.91
							975		
							787.5		
7	DW 108	primary	Nil	0	6.89	Nil	0	DENV 1–1,DENV 1–11, DENV 1–16, DENV 1–20, DENV 2–1, DENV 2–11, DENV 2–17, DENV 2–18, DENV 2–33, DENV 3–3, DENV 3–11, DENV 3–21 DENV 3–28, DENV 4–5, DENV 4–10, DENV 4–12, DENV 4–19	29.11
8	DW 138	primary	Nil	0	1.70	DENV 1–16, DENV 1–20	987.5	DENV 1–1,DENV 1–11, , DENV 2–1, DENV 2–11, DENV 2–17, DENV 2–18, DENV 2–33, DENV 3–3, DENV 3–11, DENV 3–21 DENV 3–28, DENV 4–5, DENV 4–10, DENV 4–12, DENV 4–19	27.79
							750		
9	DW 1157	primary	Nil	0	1.24	DENV1 11	775	DENV 1–1, DENV 1–16, DENV 1–20, DENV 2–1, DENV 2–11, DENV 2–17, DENV 2–18, DENV 2–33, DENV 3–3, DENV 3–11, DENV 3–21 DENV 3–28, DENV 4–5, DENV 4–10, DENV 4–12, DENV 4–19	30.39
10	DW1306	primary	Nil	0	7.73	DENV 1–20	6762.5	DENV 1–1,DENV 1–11, DENV 1–16, DENV 2–1, DENV 2–11, DENV 2–17, DENV 2–18, DENV 2–33, DENV 3–3, DENV 3–11, DENV 3–21 DENV 3–28, DENV 4–5, DENV 4–10, DENV 4–12, DENV 4–19	30.23
11	DW 198	primary	Nil	0	3.42	DENV 1–1, DENV 1–11, DENV 1–16	250	DENV 1–20, DENV 2–1, DENV 2–11, DENV 2–17, DENV 2–18, DENV 2–33, DENV 3–3, DENV 3–11, DENV 3–21 DENV 3–28, DENV 4–5, DENV 4–10, DENV 4–12, DENV 4–19	28.20
							137.5		
							375		
12	DW 1004	primary	Nil	0	1.44	DENV 3–21	450	DENV 1–1,DENV 1–11, DENV 1–16, DENV 1–20, DENV 2–1, DENV 2–11, DENV 2–17, DENV 2–18, DENV 2–33, DENV 3–3, DENV 3–11, DENV 3–28, DENV 4–5, DENV 4–10, DENV 4–12, DENV 4–19	25.35

In summary, using this T cell assay we found that none of the DENV seronegative individuals responded to these peptides and 97/105 (92.4%) of those were seropositive responded. In addition, 6/7 seronegative individuals who later developed a primary dengue infection only responded to peptides of one DENV-serotype and five individuals who previously only responded to one serotype, responded to an additional serotype after developing DHF. The seronegative individual who developed two episodes of DHF responded to two different serotypes. Therefore, this novel T cell assay appeared to be a useful tool in determining the past infecting serotype.

### Serotype-specific dengue immune responses and disease severity

**Fig 2:**
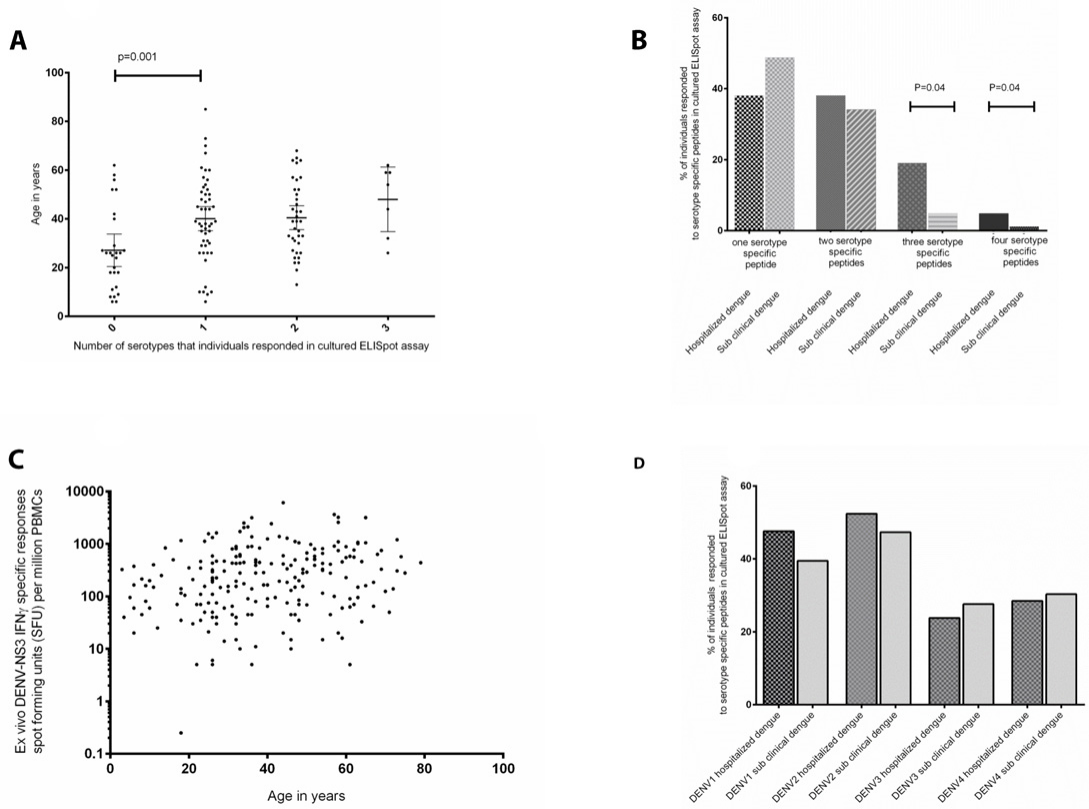
Serotype specific dengue immune responses and disease severity. a) The association of the number of DENV serotypes individual responded to with age of the individual assessed by cultured ELISpot assays. b) The numbers of responses to DENV serotype specific peptides of those who were hospitalized due to dengue and those with past mild/sub clinical dengue infection assessed by cultured ELISpot assays. c) The association of DENV-NS3 specific IFNγ-producing ex vivo T cell responses with age (Spearmans r = 0.05, p = 0.42). d) Percentage of individuals who had been hospitalized due to dengue and those with past mild/sub clinical dengue infection who responded to different serotypes of the DENV assessed by cultured ELISpot assays.

Since this novel T cell assay appeared to be a useful tool in determining the past infecting serotype, we used this assay to determine the past infecting serotypes in our DENV-seropositive cohort. As expected we found that the number of DENV serotypes that individuals responded to significantly increased with age (p = 0.001) (Fig 2A), suggesting that multiple infections are more frequent as individuals aged. In addition, as expected, individuals who were hospitalized due to dengue were more likely to be infected with multiple DENV-serotypes when compared to those who had a sub clinical dengue infection (Fig 2B). For instance, 12 (57.1%) of those who were hospitalized due to dengue responded to more than one DENV-serotype, whereas only 36 (47.3%) of those who had a past mild/sub clinical dengue infection responded to more than one. In addition, those who were hospitalized due to dengue were significantly more likely to respond to peptides from three or more DENV serotypes (p = 0.04, odds ratio 4.44, 95% CI 1.15 to 17.18).

We next proceeded to determine the frequency of DENV-NS3 specific T cell responses with infection with multiple DENV-serotypes. Although, infection with multiple DENV-serotypes increased with age, DENV-NS3 specific IFNγ responses did not increase significantly with age (Spearmans r = 0.05, p = 0.42) (Fig 2C). There was no difference in the frequency of DENV-NS3 specific IFNγ ELISpot responses in those who were hospitalized due to dengue who were likely to have had a primary dengue infection (responding to serotype specific peptides of only one DENV serotype) when compared to those who had a secondary dengue infection (those who responded to peptides of more than one serotype). No difference was again seen in individuals who had a past mild/subclinical infection which was probably a primary infection, when compared to those who had a secondary infection (those who responded to peptides of more than one serotype).

All four DENV-serotypes have known to cause severe clinical disease [39, 40]. We investigated if the type of infecting DENV serotype determined the severity of dengue infection (Fig 2D). Although those who were hospitalized due to dengue (47.6%) were more likely to respond to peptides of DENV-1 (suggestive of a past infection with DENV-1), when compared to those with past mild/sub clinical dengue infection (39.5%), the frequency of infection with DENV-1 or any other DENV serotype was not significantly different between those who were hospitalized when compared to those who had a mild/sub clinical dengue infection (p = 0.6, Odds ratio = 1.4).

## Discussion

Although DENV-specific T cells have been implicated in severe clinical disease [10, 12, 34, 35], more recent studies have shown that T cells are likely to have a protective role [13]. Extensive analysis of T cell responses by Weiskopf et al in individuals naturally infected with the DENV showed that the T cell responses of higher magnitude and which were multifunctional were directed towards HLA alleles associated with reduced disease susceptibility[13]. It was shown that DENV specific memory T cells of approximately 17% of naturally infected individuals produced 3 cytokines whereas approximately 50% were double positive. However, since the clinical disease severity in those who were naturally infected were not known in the study done by Weiskopf et al, in our study we aimed to determine the functionality of DENV-specific T cell responses in those with varying severity of past infection in a large cohort of naturally infected individuals.

Similar to the observations made by Weiskopf et al, we too found that DENV-NS3 specific T cells of 10–17% of individuals produced 3 cytokines and 29–34% of individuals produced 2 cytokines. However, we found that while DENV-NS3 specific T cells of those with past mild/sub clinical dengue infection were more likely to produce only granzyme B, those who were hospitalized due to dengue were more likely to have DENV-NS3-specific T cells that produce TNFα and IFNγ or TNFα alone. In addition, we also found that although the magnitude and the frequency of DENV-NS3 specific T cell responses were not different in those with hospitalized and mild/sub clinical dengue infection, T cells of those with past mild/sub clinical dengue were more likely to produce IFNγ. Therefore, although multiple cytokine producing T cells were observed in both group of individuals, the type of cytokines produced appear to influence the outcome of clinical disease severity rather than the frequency of DENV-NS3 specific T cells. Virus specific CD8+ T cells have shown to consist of a heterogenous population and they are thought to adjust the type of cytokine/cytokines they produce depending on the challenge [41]. Therefore, granzyme B expressing T cells are more likely to be cytotoxic than T cells expressing either TNFα or IFNγ or are double positive for these cytokines [41, 42]. Since loss of CD28 has shown to associate with acquisition of granzyme B and better cytotoxic potential [42], it would be important to study DENV specific different effector memory and central memory T cell subsets in relation to disease severity and protection in individuals naturally infected with dengue.

In this study we used DENV-NS3 peptides to determine DENV-NS3 specific T cell responses. Although NS3 is one of the most immunodominant proteins, it is not equally recognised by T cells of all naturally infected individuals [13, 32, 43]. Therefore, it would be important to investigate T cell responses to other non-structural proteins to get a more detailed overview of memory T cell responses in relation to past clinical disease severity. Since DENV-NS3 stimulates both CD4+ and CD8+ T cell responses, the cytokines produced by the T cells are likely to be from both subset of T cells. However, since CD8+ T cells are more likely to be cytotoxic and more likely to produce granzyme B than CD4+ T cells, it would be important to investigate the differences in both CD4+ and CD8+ memory T cell responses in patients with varying severity of past dengue infection.

Currently the PRNT is used in many dengue vaccination trials and epidemiological studies to determine the past infecting serotype and protection against a particular serotype [8, 24, 44]. However, it was recently shown that that individuals who demonstrated high neutralizing antibodies for a particular DENV serotype suggestive of past infection with that serotype, later went on to develop DHF when infected with the same serotype [28]. Therefore, the presence of high titres of neutralizing antibodies against a particular DENV serotype as measured by the PRNT, does not appear to reliably indicate past infection with that serotype. Since it would be important to determine the past infecting serotype for determining correlates of protection and also in dengue vaccine trials, we proceeded to investigate the usefulness of a novel T cell based assay to determine the past infecting serotype. Using a panel of previously defined peptides for the four DENVs we have further investigated the usefulness of this novel T cell based assay in determining the past infecting DENV serotype [29]. 92.4% of DENV seropositive individuals responded to at least one DENV-serotype-specific peptide in this assay, whereas none of the DENV seronegative individuals responded (N = 30).

During the study period, 7 previously seronegative individuals (who had no responses to any of the peptides) and 5 previously seropositive individuals who responded to only one serotype by this T cell assay developed DHF. Of the previously seronegative individuals, who did not respond to any of the peptides in this assay, 6/7 responded to peptides from only one DENV serotype following acute primary DHF. Of the 5 DENV seropositive individuals who responded to peptides of only one DENV serotype previously (suggestive of a previous primary dengue infection), responded to only one more additional DENV serotype following an acute hospitalized dengue infection. Therefore, 11/12 individuals who developed DHF during the study period were found to respond to at least one peptide of another DENV serotype, for which they did not respond at the time of recruitment. Although documenting the infecting DENV-serotype during acute infection would have further strengthened these findings, we did not have the access to blood sample during the acute illness. Based on epidemiological data during this time period, DENV-1 was the predominant circulating serotype and was the cause of infection in over 90% of cases (personal communication from Dr. Hasitha Tissera, Sri Lanka Epidemiology unit, Ministry of Health). Therefore, the fact that 8/12 individuals who developed DHF during the study period responded to DENV1, is compatible with the epidemiological data of DENV-1 being the predominant circulating virus serotype, although we did not confirm the serotype during the acute illness due to the unavailability of the samples during the acute illness

The PRNT which measures the neutralizing ability of antibodies is known to be influenced by the cross reactivity between different flavi-viruses [45, 46]. DENV-specific T cells have also shown to highly cross react with other flaviviruses [11]. Flaviviruses such as Japanese encephalitis virus (JEV) co-circulate in the same geographical region [47] and many countries universally vaccinate all children with the JEV vaccine. Therefore, in assessing the burden of dengue and in determining the past infecting DENV serotype, it is crucial that such an assay does not cross react with other flavi-viruses. In this novel T cell assay, none of dengue seronegative individuals who had received the JEV vaccine responded to any of the DENV peptides. Therefore, this assay does not appear to pick up JE specific cross reactive T cell responses. This is a further added advantage over the PRNT, where low titres of neutralizing flavi-virus antibodies are detected in dengue seronegative individuals due to cross reactivity of the flavi-virus antibody responses[23, 24, 48].

Since this novel T cell based assay appeared to be a useful tool in determining the past infecting DENV serotype, we used this assay to determine the association of past infecting serotype and clinical disease severity. As expected, those with past hospitalized dengue were more likely to respond to more than one DENV serotype suggesting that they had hospitalized dengue due to a secondary dengue infection. However, 37.8% of those with past mild/sub clinical dengue infection responded to 2 serotypes and 5.4% responded to 3 serotypes suggesting that mild/sub clinical dengue infection is also common with both secondary and tertiary dengue infections. Interestingly, the number of past dengue infections did not appear to influence the frequency of DENV-NS3 specific IFNγ responses as the frequency and magnitude of responses were similar in individuals who responded to one, two or three DENV serotypes.

In summary, the majority of individuals naturally infected with the DENV, had DENV-NS3 specific T cell responses, which produce multiple cytokines. However, DENV-NS3 specific T cells of those who had a past mild/sub clinical dengue infection were more likely to produce only granzyme B, whereas, T cells of those with past hospitalized dengue infection were more likely to be double positive for IFNγ and TNFα. In addition, we have also investigated the usefulness of a novel T cell based assay, which can be used to determine the past infecting DENV serotype. Since the peptides used in this assay do not appear to be cross reactive with JEV and since those who develop primary and secondary dengue appear to be making expected responses to these peptides, it is likely to be useful in determining correlates of protection in large epidemiogical studies and in dengue vaccine trials.

## Supporting Information

S1 Checklist Strobe checklist. (DOCX)
